# A CTD–Pfizer collaboration: manual curation of 88 000 scientific articles text mined for drug–disease and drug–phenotype interactions

**DOI:** 10.1093/database/bat080

**Published:** 2013-11-28

**Authors:** Allan Peter Davis, Thomas C. Wiegers, Phoebe M. Roberts, Benjamin L. King, Jean M. Lay, Kelley Lennon-Hopkins, Daniela Sciaky, Robin Johnson, Heather Keating, Nigel Greene, Robert Hernandez, Kevin J. McConnell, Ahmed E. Enayetallah, Carolyn J. Mattingly

**Affiliations:** ^1^Department of Biological Sciences, 3510 Thomas Hall, North Carolina State University, Raleigh, NC 27695-7617, USA, ^2^Computational Sciences Center of Emphasis, 200 Cambridgepark Drive, Pfizer Inc., Cambridge, MA 02139, USA, ^3^Department of Bioinformatics, P.O. Box 35, Old Bar Harbor Road, MDI Biological Laboratory, Salisbury Cove, ME 04672, USA, ^4^Compound Safety Prediction, MS 8118-B3, Eastern Point Road, Pfizer Inc., Groton, CT 06340, USA, ^5^Computational Sciences Center of Emphasis, Pfizer Inc., Ramsgate Road, Sandwich, Kent CT13 9NJ, UK, ^6^Computational Sciences Center of Emphasis, 558 Eastern Point Road, Pfizer Inc., Groton, CT 06340, USA and ^7^Drug Safety Research and Development, 558 Eastern Point Road, Pfizer Inc., Groton, CT 06340, USA

## Abstract

Improving the prediction of chemical toxicity is a goal common to both environmental health research and pharmaceutical drug development. To improve safety detection assays, it is critical to have a reference set of molecules with well-defined toxicity annotations for training and validation purposes. Here, we describe a collaboration between safety researchers at Pfizer and the research team at the Comparative Toxicogenomics Database (CTD) to text mine and manually review a collection of 88 629 articles relating over 1 200 pharmaceutical drugs to their potential involvement in cardiovascular, neurological, renal and hepatic toxicity. In 1 year, CTD biocurators curated 2 54 173 toxicogenomic interactions (1 52 173 chemical–disease, 58 572 chemical–gene, 5 345 gene–disease and 38 083 phenotype interactions). All chemical–gene–disease interactions are fully integrated with public CTD, and phenotype interactions can be downloaded. We describe Pfizer’s text-mining process to collate the articles, and CTD’s curation strategy, performance metrics, enhanced data content and new module to curate phenotype information. As well, we show how data integration can connect phenotypes to diseases. This curation can be leveraged for information about toxic endpoints important to drug safety and help develop testable hypotheses for drug–disease events. The availability of these detailed, contextualized, high-quality annotations curated from seven decades’ worth of the scientific literature should help facilitate new mechanistic screening assays for pharmaceutical compound survival. This unique partnership demonstrates the importance of resource sharing and collaboration between public and private entities and underscores the complementary needs of the environmental health science and pharmaceutical communities.

Database URL: http://ctdbase.org/

## Introduction

Manual curation of the scientific literature is a specialized endeavor that transforms authors’ free-text information into annotated knowledge, via the use of controlled vocabularies and ontologies, by professional biocurators (1–2). This process helps standardize, harmonize and organize disparate data from scientific publications into a structured format, making it more manageable and computable for analysis.

Safety researchers from Pfizer Inc., the world's largest research-based drug company (3), set out to leverage decades’ worth of toxicity data from the published literature to help build a comprehensive database of drug–event relationships. A critical feature of drug development is pharmaceutical compound survival, wherein new molecular entities are allowed to continue through clinical development by demonstrating positive efficacy as well as safety (4). Advanced screening methods can improve early detection of safety issues during compound development; however, a comprehensive reference set of molecules with well-defined toxicities is vital for training and validation purposes, as this defines the confidence in being able to apply new assays or technologies to safety assessment. In addition, the availability of high quality and extensive adverse drug event annotations is critical for generating novel hypotheses that can facilitate new mechanistic screening assays. Unfortunately, public resources of drugs and their side effects amenable to computational methods are limited. DrugBank, a comprehensive database for therapeutic drug information (5), provides side effects only as brief free-text without references. The Food and Drug Administration hosts the Adverse Event Reporting System (FAERS), where drug makers, prescribers and consumers can submit reports of drug-induced side effects, but sophisticated data mining algorithms are required to detect safety signals before they are reported in the literature (6–7). SIDER mines drug labels to create a database of drugs, side effects and side effect frequency (8). However, neither of these last two sources takes advantage of the scientific literature, in which drug-induced phenomena are documented in a variety of settings, such as *in vitro* and *in vivo* methods, across species, for approved indications, off-label uses and for drugs in development.

To aid Pfizer safety researchers in the development of a comprehensive database for literature-based drug-induced events, a collaboration was initiated with the staff at the Comparative Toxicogenomics Database (CTD), a public database that promotes understanding about how the molecular interactions between environmental chemicals and genes affect human health (9–11). CTD biocurators have extensive expertise in reviewing the peer-reviewed scientific literature and manually curating a triad of chemical–gene, chemical–disease and gene–disease interactions (12–14). CTD software engineers integrate these data with each other and with external datasets to generate novel inferences between chemicals, genes, diseases, Gene Ontology (GO) annotations and pathways; predict molecular pathways affected by chemical exposures; and identify similar chemicals and diseases based on shared toxicological profiles (15–17).

CTD has historically focused on a broad range of environmental chemicals, including arsenic (12), tetrachlorodibenzodioxin (13), bisphenol A (15) and heavy metals (18). In order to direct curation to pharmaceutically relevant articles, Pfizer scientists designed text-mining strategies to generate a set of over 88 000 research articles enriched for drugs of therapeutic interest and their effects on cardiovascular, neurological, renal and hepatic systems. In 1 year, CTD biocurators used existing strategies and tools to manually curate chemical–gene, chemical–disease, gene–disease, chemical–phenotype and gene–phenotype interactions.

The collaboration generated over 2 50 000 manually curated interactions for chemical-induced events. CTD has integrated this information with its public website (http://ctdbase.org/), while Pfizer has combined these data with internal databases to help test and evaluate compound safety. The collaboration has greatly enhanced and supplemented CTD public content by the addition of this drug-related information. Further expansion and integration of the phenotype data with CTD is a future goal.

## Methods

### Nota bene

1. Pfizer studies and develops ‘drugs’, while CTD curates ‘chemicals’ using a controlled vocabulary that is a modified subset of the ‘Drugs and Chemicals [D]’ branch from Medical Subject Headings (MeSH); this CTD vocabulary includes environmental chemicals as well as pharmaceuticals (12). For all intents and purposes, the words ‘drug’ and ‘chemical’ should be considered interchangeable in this report.

2. CTD distinguishes between ‘diseases’ and ‘phenotypes’ wherein phenotype refers to a non-disease-term biological event. For example, ‘abnormal cell proliferation’ is a phenotype while ‘lung cancer’ is a disease, ‘increased adipogenesis’ is a phenotype while ‘obesity’ is a disease and ‘decreased blood pressure’ is a phenotype, while ‘idiopathic orthostatic hypotension’ is a disease. For disease terms, CTD used the MEDIC vocabulary (19), and for phenotypes used 143 terms from the ‘Phenomena and Process [G]’ branch of MeSH (preselected by Pfizer).

### Pfizer text-mining strategy

Previously, an in-house effort at Pfizer to develop a drug safety database provided an initial gold standard of 3 017 relevant articles representing 5 species and 650 unique drugs of importance to Pfizer. Pfizer scientists annotated these articles to a hand-selected set of safety findings. Pfizer analysed the collection with two aims: develop queries to automatically extract drug–adverse event relationships and incorporate publicly available controlled vocabularies to facilitate integration with other data sources. To automate extraction of drug-adverse event relationships, the articles were analysed for frequently occurring semantic patterns relating drugs to adverse event terms. As well, MeSH and their qualifiers (which are used to index safety findings by the National Library of Medicine) were also analysed for possible utility. A mix of high-precision and high-recall patterns was selected and implemented for querying (Table 1) using Linguamatics I2E software (Linguamatics, Cambridge, UK). Safety terms were also derived from the MeSH ‘Diseases [C]’ branch, focusing on the sub-branches of cardiovascular, neurological, hepatic and renal diseases. Applying the text-mining strategy to abstracts in Medline identified 78 263 articles, henceforth referred to as the *drug–disease corpus*. Queries Q1, Q2 and Q3 retrieved 57, 33 and 10% of the text-mined statements for this corpus, respectively (Table 1).

**Table 1.** Pfizer's text-mining queries

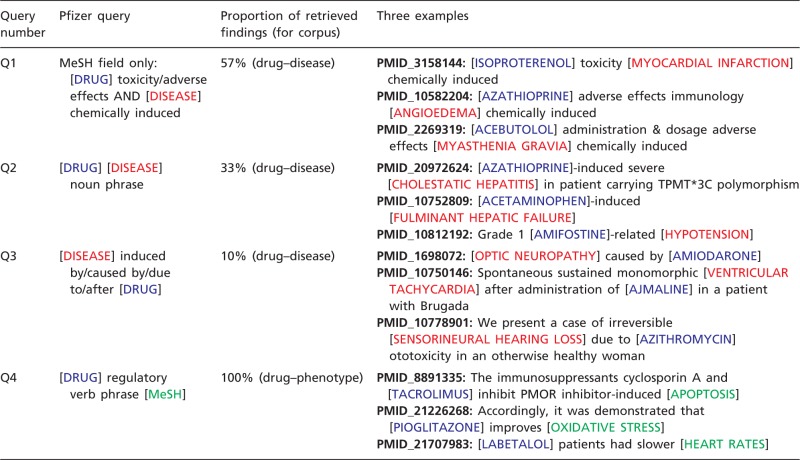

To identify phenotype (non-disease) concepts of interest, Medline records from the 3 017 articles were queried using Pfizer’s own disease dictionary. Articles with no disease matches were further analysed by reviewing their associated MeSH terms and the semantic patterns that related drugs to drug-induced phenomena. Frequently occurring MeSH terms from the ‘Phenomena and Processes [G]’ branch were selected for drug–phenotype event retrieval. After trying various combinations of drug and MeSH term patterns, the best precision/recall balance was achieved with the semantic pattern: ‘[DRUG] regulatory verb phrase [MeSH]’ in an ordered phrase with no more than two words between the bracketed concepts (query Q4, Table 1). The MeSH ‘Anatomy [A]’ branch was included as an optional query element to assist CTD biocurators with capturing tissues when available. A set of query terms representing five species of interest (human, mouse, rat, non-human primate and dog) were an additional required feature that could appear anywhere in the Medline record. This text-mining strategy applied to abstracts collated 10366 articles, henceforth called the *drug–phenotype corpus*. Query Q4 retrieved 100% of the text-mined statements for this corpus (Table 1).

In total, Pfizer provided CTD with 88 629 text-mined articles (based upon abstracts). These articles were derived from 4 729 journals published over 66 years (from 1945 to 2011), evincing a broad and robust coverage of the literature.

### CTD curation strategy

CTD agreed to complete the curation in 1 year. To accomplish that goal, we first tested a sample of articles provided by Pfizer to estimate time duration and biocurator needs. This test set (85 articles) was 55% curatable, but had a much faster review rate (4.2 min per article) than typical CTD collections (∼20 min per article). This increased rate was attributed to the articles’ content, which consisted predominantly of chemical–disease information, rather than chemical–gene information. Based on this pilot experiment, it was estimated that 5 full-time biocurators could process 70–100 articles per day per biocurator to reach a projected goal of ∼100 000 articles in 12 months. In October 2010, a specialized pharma-edition of CTD’s *Curation Manual* was written and five professional biocurators were hired and underwent intensive and detailed on-site training at CTD. Although each biocurator worked remotely subsequent to the training, mechanisms were in place to facilitate communication, answer questions and resolve policy issues. As well, CTD launched a web-based Curation Tool designed to expedite work, centralize and consolidate biocuration activities, eliminate the use of Excel spreadsheets and facilitate quality control (20).

To ensure goals were met during the project timeline, CTD biocurators submitted biweekly invoices that recorded the number of hours worked and the number of articles completed. These invoices were used to calculate review rate metrics to help monitor the progress of the project by dividing the total billing time by the total number of articles reviewed during the billing period. Review rates calculated from such reports were ‘macro’ rates and represented an upper-bound estimate that reflected the true cost of curation with overhead, since the amount of total time billed incorporated time for other daily tasks besides just curation (such as exchanging emails, reviewing work, participating in monthly phone conferences, etc.).

### Curation pipeline

The drug–disease corpus (78 263 articles) was parceled into 4 files according to the system-of-interest text mined by Pfizer: 22 651 articles (cardiovascular), 42 311 articles (neurological), 13 131 articles (renal) and 6 277 articles (hepatic), with many articles overlapping for more than 1 disease category. These 4 files were equally distributed to 5 CTD biocurators who were provided with the PubMed identification number (PMID), the Pfizer-triaged drug term(s) and the Pfizer-triaged disease term(s) for each article. These articles were curated only for chemical–disease, gene–disease and chemical–gene interactions; they were not curated for phenotype data (except for an incidental 401 articles during the transition phase to the drug–phenotype corpus). The drug–phenotype corpus (10 366 articles) contained the article PMID, and Pfizer-triaged terms for drug, phenotype, species and anatomy. This collection was also evenly divided among the CTD biocurators and curated for relevant phenotype data, as well as any chemical–disease, gene–disease and chemical–gene interactions described.

Biocurators followed CTD’s well-documented curation process (9–12, 20). Briefly, biocurators performed six tasks: recorded whether the article should be curated; curated articles following CTD’s policies (which included curating every mentioned chemical, gene or disease, not just the terms for which an article was triaged); captured organism’s taxon for each interaction; indicated whether interactions were garnered from the abstract or full text; recorded whether interactions were studied *in vitro* or *in vivo*; and indicated whether an interaction was derived from a high-throughput assay. Biocurators curated from just the abstract whenever possible, but examined the full text if necessary to resolve any relevant issues mentioned in the abstract.

Chemical–gene interactions were composed by biocurators selecting from over 50 action codes that could be multiplexed to describe detailed events. Disease curation, on the other hand, had a more streamlined process, in that only a binary relationship was established between a chemical/gene and a disease using two available codes: ‘M’ to describe a mechanistic or marker relationship to a disease or ‘T’ to describe a known or potential therapeutic relationship to a disease. All data were publicly released to CTD users on 9 January 2012.

### Constructing –Tox and –Treat dataset profiles

CTD’s Batch Query tool (http://ctdbase.org/tools/batchQuery.go) was used to retrieve datasets on 17 July 2013 (CTD version 13 268) of all curated chemicals associated with all curated diseases representing cardiovascular toxicity (CardioTox), neurological toxicity (NeuroTox), renal toxicity (RenalTox) and hepatic toxicity (HepatoTox). Our hierarchical disease vocabulary MEDIC allows annotated data from child pages to be subsumed to parent pages (19). Thus, CTD’s Cardiovascular Diseases page reports chemicals annotated to this term as well as chemicals curated to disease descendants (e.g., hypertension, long QT syndrome, angiodema, etc.). Here, we used the parent terms Cardiovascular Diseases for CardioTox, Nervous System Diseases for NeuroTox, Kidney Diseases for RenalTox and Liver Diseases for HepatoTox. Results were downloaded and sorted to retrieve chemicals with a ‘marker/mechanism’ relationship (–*Tox* profiles); chemicals with a ‘therapeutic’ relationship were used to construct the treatment profiles (e.g., CardioTreat, NeuroTreat, RenalTreat and HepatoTreat). The data were derived from chemicals and diseases curated from the Pfizer drug–disease corpus and incidental data curated from other CTD projects. A complete list of diseases and chemicals for the four toxicity profiles is provided in Supplementary File 1.

### CTD phenotype curation

The drug–phenotype corpus was manually curated for chemical–gene–disease, chemical–phenotype and gene–phenotype interactions. To capture this new data, the Curation Tool was modified to accommodate phenotype terms. Phenotype interactions were annotated to a taxon, and biocurators also curated anatomical terms to describe where the phenotype occurred. When necessary, multiple anatomical terms were concatenated to increase specificity (e.g., Brain—Blood Vessels—Endothelial Cells). For this pilot project, CTD used MeSH terms as a source for both phenotype and anatomy controlled vocabularies (21). For phenotypes, we required a control vocabulary of non-disease-term biological events that was species independent. This ruled out several well-established disease vocabularies and organism-specific phenotype ontologies. Instead, Pfizer scientists selected 143 terms from the MeSH ‘Phenomena and Processes [G]’ branch to be used as phenotype terms, as these best reflected Pfizer’s interest. For an anatomical controlled vocabulary, biocurators selected from 2 774 terms from the MeSH ‘Anatomy [A]’ branch, which provided a deep and robust coverage of body systems and cell types. This dataset is not yet integrated with CTD’s dynamic web-based content, but all phenotype interactions are publicly available by clicking on http://ctdbase.org/reports/CTD_pheno_ixns.xls to download an Excel file (6.6 MB).

### Phenotype–disease inference analysis

Chemicals that were annotated to both phenotypes and diseases were used to make inferred relationships between phenotypes and diseases. Data from the chemical–phenotype file was integrated with CTD’s public chemical–disease dataset on 8 May 2013 (CTD version 13 096) to generate 102828 phenotype–disease inferences (Supplementary File 2). This file was then restricted to inferences with 10 or more shared chemicals to increase the stringency of the inferred relationship. A matrix of the number of shared chemicals (log2-transformed) for 74 phenotypes × 750 diseases was constructed and analysed as a two-dimensional hierarchical clustering and rendered as a heatmap using JMP version 8.0 (SAS Institute, Cary, NC). For the clustering, distance was calculated using Ward’s minimum variance method with standardized values (22). The dendrogram was manually trimmed to represent the overall similarity among diseases and identified 18 clusters. For disease classification, all 750 diseases were mapped to 36 generic disease categories using CTD’s MEDIC-Slim disease vocabulary (9) to better summarize and visualize the disease classifications, as previously described (18). The top four disease classes, as a percentage of each cluster, were graphed as a pie chart.

### Calculating text-mining precision

Pfizer text mining, using internal dictionaries, retrieved 2 310 normalized concepts (1 261 drug, 958 disease and 91 phenotype terms). The precision of term and event (e.g., chemical–disease or chemical–phenotype) extraction was measured by comparing the text-mined terms supplied in the Pfizer corpus (input) to the CTD curation dataset (output), calculating precision at both the article level (PMID, PubMed identification number) and at the term level for each individual corpus as well as the combined corpus, and for each individual term as well as all terms in aggregate. For the article-level metrics, two scores were calculated: one comparing the number of hits against all the articles in each text-mined corpus (TM-All) and another against solely the curated articles in each corpus (TM-Curated). Although the vast majority of terms used by the Pfizer text-mining processes were directly resolvable to their counterpart CTD chemical, disease and phenotype controlled vocabularies, cross references were created by CTD staff to resolve many of the remaining unmatched terms (e.g., the Pfizer text-mining term ‘Retts Disease’ was mapped to CTD’s MEDIC disease term ‘Rett Syndrome’). Of the 2310 total Pfizer text-mining terms, 142 (6%) were irresolvable by CTD and dropped from this analysis; these included 70 disease terms (e.g., suicidal behavior, hunger, ego, emotions, etc.) and 72 drug terms (e.g., uk-008451, DRUG430730, immune globulin, fb-532, etc.). If a Pfizer text-mined term (input) was ultimately curated by CTD (output) for the respective PMID to a CTD counterpart term, resolvable synonym to the counterpart term or a child of the counterpart term, it was counted as a true positive; if CTD curated to unrelated chemicals or diseases, the Pfizer terms were scored as false positives.

## Results and discussion

### CTD curation metrics

**Figure 1.**
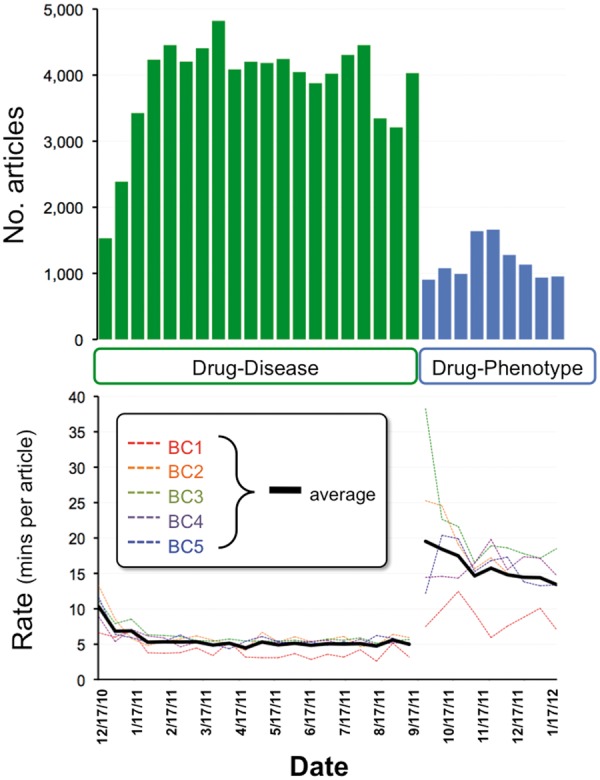
Project metrics. From December 2010 to September 2011, five CTD biocurators reviewed 78 263 articles for drug–disease information (top graph, green bars). Biocurators curated from just the abstract whenever possible, but examined the full text if necessary to resolve any relevant issues mentioned in the abstract. Review rates for each individual biocurator (bottom graph, BC1–BC5, dotted colored lines) were calculated based upon billing invoices, and the biweekly average of all five biocurators is also shown (solid black line). In September 2011, biocurators transitioned to reviewing 10366 articles for drug–phenotype information (top graph, blue bars). An increase in performance (as reflected by a decrease in rate) is seen as both projects progressed. For drug–disease curation, the average rate initiated at 10.3 min per article (17 December 2010) and ultimately improved to an average rate of 5.5 min per article over the entire period. For drug–phenotype curation, the average initial rate was 19.5 min per article (17 September 2011), improving to 13.4 min per article (13 January 2012), with an aggregate average rate of 15.9 min per article over the period.

For the drug–disease corpus (78263 articles), 5 CTD biocurators reviewed the set in ∼9 months and averaged 5.5 min per article over the entire project period (Figure 1). This fast review rate was due to the articles being intentionally skewed more toward literature describing binary chemical and disease relationships, as opposed to gene information that tends to be more complex. The average number of curated chemical, disease and gene terms per curated article were 1.7, 2.0 and 0.5, respectively (Table 2), demonstrating ∼4-fold predominance of chemical and disease terms compared to genes. Curating gene information is the most time-consuming aspect of CTD biocuration because it typically requires access to the full text to resolve species information and the official gene symbol (using synonyms, alternative names, reactive monoclonal antibodies, DNA sequences, derived RT-PCR primers, accession identifiers or citations mentioned by the authors). Constructing chemical–gene interactions also takes longer to code because of the option of over 50 different action codes that can be multiplexed into detailed, nested structured notations (20). On the other hand, resolving chemical and disease terms to their official controlled vocabularies is often accomplished quickly and easily from the title or abstract, and the structured notation for such interactions is exclusively binary (20).

**Table 2.** Article and interaction statistics

Data	Drug–disease corpus	Drug–phenotype corpus	Total
No. articles reviewed	78 263	10 366	88 629
No. articles curated^a^	51 884	9 646	61 530
No. articles rejected	26 379	720	27 099
No. total interactions	183 849	70 324	254 173
No. chemical–disease interactions	145 366	6 807	152 173
No. chemical–gene interactions	32 539	26 033	58 572
No. gene–disease interactions	4 603	742	5 345
No. phenotype interactions	1 341^b^	36 742	38 083
Average no. interactions per curated article	3.5	7.3	n/a
Average no. chemicals per curated article	1.7	2.3	n/a
Average no. diseases per curated article	2.0	0.5	n/a
Average no. genes per curated article	0.5	1.9	n/a
Average no. phenotypes per curated article	<0.0^b^	1.6	n/a
Average no. anatomy terms per curated article	<0.0^b^	1.7	n/a
Average no. taxa per curated article	1.0	1.0	n/a

^a^abstract curation whenever possible; full text was examined if necessary to resolve issues.

^b^curated from 401 drug–disease articles during transitional period to drug–phenotype phase.

n/a, not applicable.

For the drug–phenotype corpus (10 366 articles), the biocurators reviewed the set in 3 months and averaged 15.9 min per article, yet there appeared to be a greater degree of individual biocurator variability, ranging between 8.7 and 21.1 min per article (Figure 1). The comparatively longer time to curate this set (vs. the drug–disease corpus) and the variability is likely due to several factors. First, biocurators needed to familiarize themselves with a new curation module with two new controlled vocabularies (phenotype and anatomy). Second, drug–phenotype articles had an overall greater density of curatable information compared to drug–disease articles, with the former averaging 7.3 interactions per curated article vs. 3.5 in the latter (Table 2). Third, drug–phenotype articles contained more gene information compared to drug–disease articles: 1.9 genes per article vs. 0.5, respectively (Table 2), and, as explained above, curating gene information tends to be more time-consuming.

For both projects, CTD biocurators curated from the abstract whenever possible, but were allowed to curate also from the full text if necessary, especially to resolve any relevant issues mentioned in the abstract. A total of 61 530 articles were manually curated: 51 884 for drug–disease and 9 646 for drug–phenotype (Table 2). For the drug–disease corpus, 40 781 (79%) were curated exclusively from the abstract and 11 103 (21%) required at least some curation from the full text. Of the 9 646 articles for the drug–phenotype corpus, 7 480 (78%) were curated solely from abstracts and 2 166 (22%) required going to the full text. At the interaction level, a total of 254 173 interactions were manually curated: 183 849 for drug–disease and 70 324 for drug–phenotype (Table 2). For the drug–disease corpus, 123 563 (67%) were from the abstract and 60 286 (33%) from the full text. For the drug–phenotype corpus, 42 044 (60%) were abstract-derived and 28 280 (40%) were garnered from the full text.

### Enhancement of CTD content

Of the 182 508 interactions curated from the drug–disease corpus, 145 366 (80%) were for chemical–disease, 32 539 (18%) for chemical–gene and only 4603 (2%) for gene–disease interactions (Table 2), reflecting the intentional and successful skewing of this corpus for drug–disease information. An additional 33 582 interactions involving chemicals, genes and diseases were also collected from the drug–phenotype corpus (Table 2). The Pfizer articles complemented CTD’s routine chemical-centric approach to article selection (14). CTD content has been primarily based upon articles triaged by querying PubMed for both a chemical-of-interest and a gene concept to bias for articles describing chemical–gene interactions. Here, however, the Pfizer drug–disease corpus was instead skewed for chemical and disease terms without the necessity of gene information, allowing for a very different type of corpus to be collated.

Of the combined 58 572 chemical–gene interactions manually curated, 52 387 (89%) were interactions not yet represented in CTD. For the 5 345 gene–disease interactions, 78% were new to CTD and for the 152 173 chemical–disease interactions, 47% were new. In total, these interactions have expanded and enhanced CTD with respect to new chemical–disease information, especially for pharmaceuticals.

**Figure 2.**
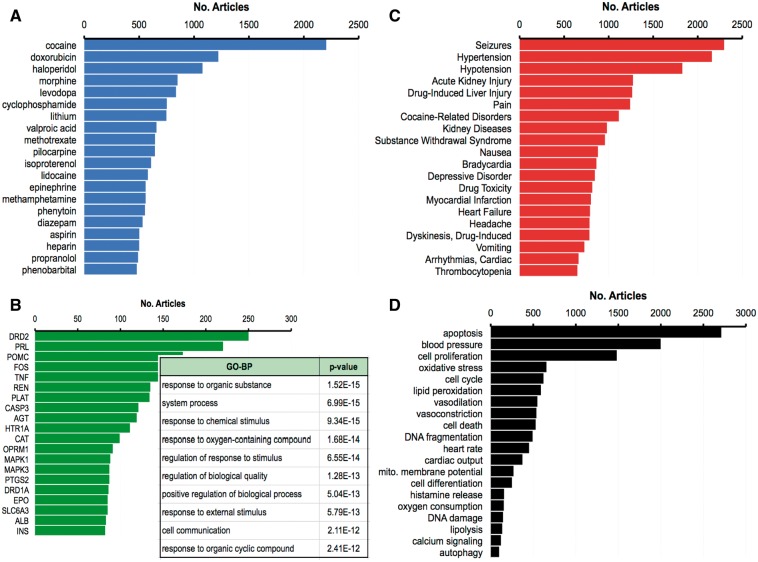
Top 20 curated terms. The 20 most frequently curated chemicals (**A**, blue), genes (**B**, green) and diseases (**C**, red) from the drug–disease corpus, as measured by the number of articles from whence the term was curated, out of 51 884 total curated articles for this corpus. The inset in (B) lists the 10 most significantly enriched GO-BP and their corrected *p*-value (Bonferroni multiple testing adjustment) for the top 20 genes. (**D**) The 20 most frequently curated phenotypes (black) from the drug–phenotype corpus (out of a total of 9646 curated articles).

From the drug–disease corpus, a total of 5 562 chemicals, 9 167 genes and 2 697 diseases were ultimately curated (reflecting CTD’s policy to curate every mentioned chemical, gene or disease, not just the terms for which an article was triaged), and the 20 most frequently curated terms for each group are shown (Figure 2A–C). Since genes were not among the text-mining selection criteria in the triaging process, they could act as an unbiased indicator of the results. As a means of gauging the type of information being captured, we evaluated the top 20 genes using CTD’s *Set Analyzer* tool (http://ctdbase.org/tools/analyzer.go) to find their associated GO biological processes (GO-BP) (Figure 2B, inset). Four of the top 10 most significant processes were types of ‘response to chemical stimulus’ (GO:0042221), including responses to organic substances (GO:0010033), oxygen-containing compounds (GO:1901700) and organic cyclic compounds (GO:0014070), supporting and confirming the curated genes’ role in toxicogenomic responses and interactions with drugs. For comparison, we sampled 800 genes (20 at a time) randomly selected from the bottom half of the 9 167 gene list; none of these gene sets had any enriched GO processes achieving a significance threshold (corrected *p*-value < 0.01; not shown).

### Toxicity profiles

This curation project expanded the number and coverage of chemical–disease interactions in public CTD, allowing a better representation of the drug-induced events for the four physiological systems central to Pfizer drug safety prediction: cardiovascular, neurological, renal and hepatic toxicity. We constructed four data profiles of chemicals with ‘mechanism/marker’ relationships to diseases related to these four systems: CardioTox (composed of 1 847 chemicals and 305 cardiovascular diseases), NeuroTox (2 533 chemicals and 522 nervous system diseases), RenalTox (1 047 chemicals and 64 kidney diseases) and HepatoTox (1 275 chemicals and 55 liver diseases). A list of the chemicals and diseases for each toxicity profile is provided in Supplementary File 1.

**Figure 3.**
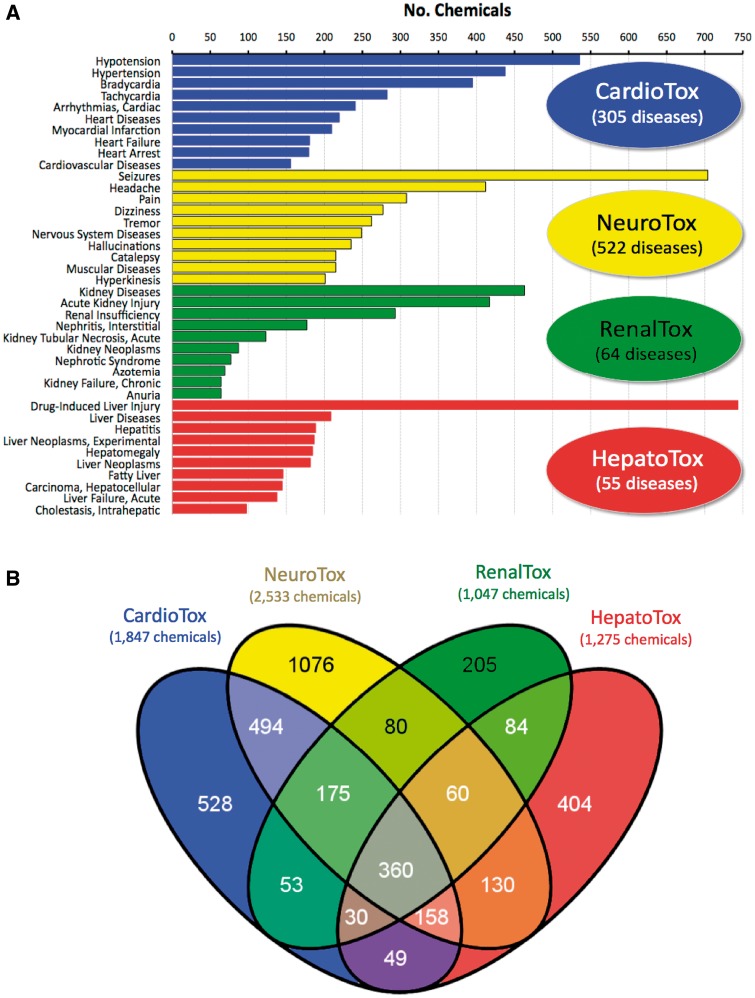
Diseases and chemicals for four system toxicity profiles. (**A**) The top 10 curated diseases are ranked by the number of chemicals curated to each disease for cardiovascular toxicity (CardioTox, blue; 305 diseases), neurological toxicity (NeuroTox, yellow; 522 diseases), kidney toxicity (RenalTox, green; 64 diseases) and liver toxicity (HepatoTox, red; 55 diseases). (**B**) Venn diagram of 3 886 chemicals associated with CardioTox (blue; 1847 chemicals), NeuroTox (yellow; 2533 chemicals), RenalTox (green; 1047 chemicals) and HepatoTox (red; 1275 chemicals). There are 360 chemicals (center gray subset) common to all four systems.

In CardioTox, the most frequently curated toxicities were for abnormal blood pressure (hypotension, hypertension) and heart rate (bradycardia, tachycardia and arrhythmias); for NeuroTox the most abundant relationship was between 704 chemicals and seizures; kidney diseases and injuries were the most common curated endpoints for RenalTox; and for HepatoTox, drug-induced liver injury was overwhelmingly represented for 744 chemicals (Figure 3A). A Venn analysis of the associated drugs showed chemicals unique to each toxicity system, chemicals common to more than one system and 360 shared chemicals that affected all 4 systems (Figure 3B). One possibility for some overlap may be due to disease terms mapping to more than one physiological system; for example, brain infarction is both a cardiovascular and a neurological disease; thus, chemicals annotated to it are automatically shared between CardioTox and NeuroTox. However, the chemical crossover between system toxicities due to shared disease ontology was limited, since only 72 diseases mapped to 2 systems, 1 disease mapped to 3 systems (Zellweger Syndrome to NeuroTox, RenalTox and HepatoTox) and no diseases mapped to all 4 systems. Thus, the majority of overlapping chemicals is due to drugs affecting multiple systems, perhaps through common genes or pathways or possibly via non-genetic modes.

These toxicity profiles can be further analysed to improve mechanistic understanding of drug-induced events. Computational toxicology methods and advanced chemoinformatics can use these curated datasets, combined with molecular structure files, to correlate structural motifs and defined toxic endpoints to identify potential alerts for chemical-associated events for cardiovascular, neurological, hepatic or renal toxicity (23,24). As well, the common 360 chemicals shared by all 4 systems might identify molecular processes and signals prevalent to many physiological systems. Additional profiles can be easily constructed for other events, such as SkinTox, CancerTox, ImmunoTox, LungTox, etc. (Table 3). Conversely, complementary treatment profiles (–*Treat*) can be constructed for drugs with a curated ‘therapeutic’ relationship (Table 3) to look for likewise connections between structural motifs and positive outcomes for diseased systems to potentially advance pharmaceutical drug design or repositioning.

**Table 3.** Chemical–disease profiles from CTD

Disease term^a^	Toxicity profile name	No. chemicals (M-type)^b^	No. diseases	Therapeutic profile name	No. chemicals (T-type)^b^	No. diseases
Cardiovascular diseases	CardioTox	1 847	305	CardioTreat	1 543	231
Nervous system diseases	NeuroTox	2 533	522	NeuroTreat	2 216	476
Liver diseases	HepatoTox	1 275	55	HepatoTreat	635	56
Kidney diseases	RenalTox	1 047	64	RenalTreat	528	62
Skin diseases	SkinTox	1 145	146	SkinTreat	667	165
Neoplasms	CancerTox	1 007	240	CancerTreat	1 516	312
Immune system diseases	ImmunoTox	982	126	ImmunoTreat	720	141
Respiratory tract diseases	LungTox	945	132	LungTreat	706	116
Metabolic diseases	MetaboloTox	855	142	MetaboloTreat	535	140
Hematologic diseases	HematoTox	822	98	HematoTreat	313	82
Gastrointestinal diseases	GastroTox	583	88	GastroTreat	538	82
Eye diseases	EyeTox	542	129	EyeTreat	281	107
Endocrine system diseases	EndoTox	522	89	EndoTreat	530	90
Muscular diseases	MuscleTox	497	46	MuscleTreat	198	33
Lymphatic diseases	LymphaTox	197	43	LymphaTreat	295	52
Bone diseases	BoneTox	148	49	BoneTreat	184	48
Connective tissue diseases	ConnectiTox	122	25	ConnectiTreat	166	40

^a^Input term used to retrieve data using CTD's Batch Query tool.

^b^M, marker/mechansim-type relationship (for –*Tox* files); T, therapeutic-type relationship (for *-Treat* files).

### Phenotype curation

**Figure 4.**
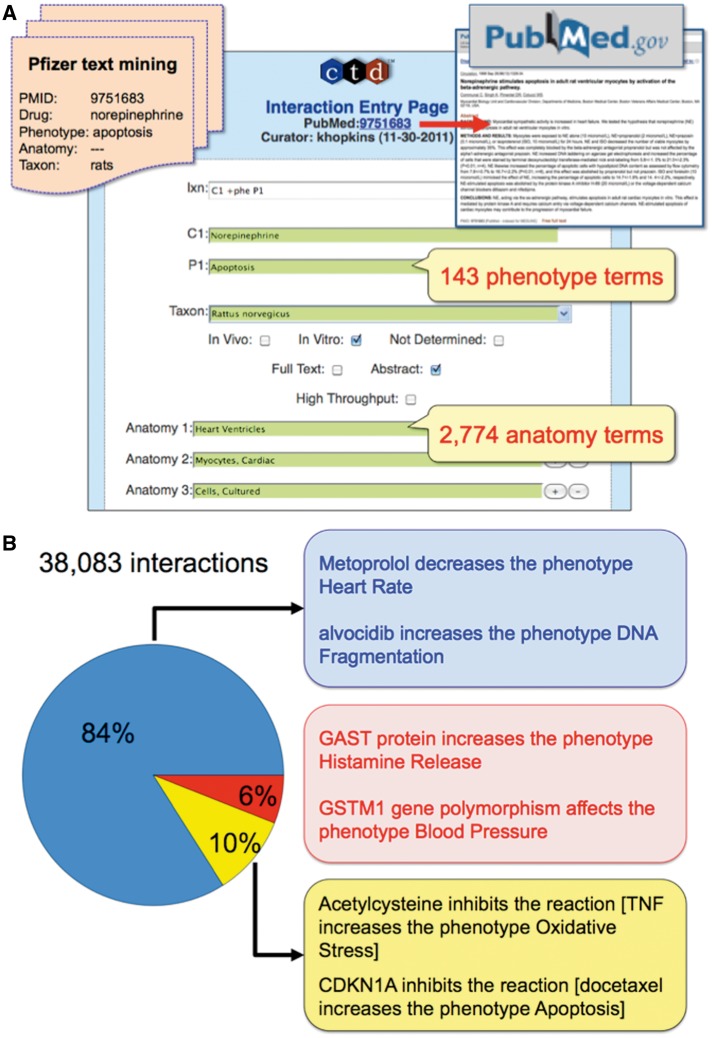
CTD’s phenotype curation module. (**A**) Pfizer provided CTD with 10 366 articles text mined for a drug-of-interest, phenotype, anatomy and taxon (orange file, upper-left corner). Biocurators entered each article’s PMID into the CTD Curation Tool and retrieved the PubMed abstract for curatorial review (red arrow and box, upper-right corner). Biocurators curated from just the abstract whenever possible, but examined the full text if necessary to resolve any relevant issues mentioned in the abstract. Drug–phenotype interactions were generated using CTD’s structured notation, codes and controlled vocabularies in the Curation Tool (blue panel). In this prototype, 143 phenotype terms and 2774 anatomy terms were available. Here, the biocurator coded an interaction (Ixn field) describing how the drug norepinephrine (C1 field) resulted in increased apoptosis (P1 field) using an *in vitro* system from rats (Taxon field) of cultured ventricular myocytes (Anatomy 1–3 fields). The Curation Tool validates terms entered by the biocurator in real-time, and the green color of the text boxes indicates the terms are valid for curation. (**B**) Examples of CTD’s curated phenotype interactions. Of the total 38 083 interactions, 84% describe chemical–phenotype interactions (blue box), 6% gene–phenotype interactions (red box) and 10% complex chemical–gene–phenotype interactions (yellow box).

Chemicals can also affect biological systems before causing a disease or without necessarily resulting in a disease. At CTD, we refer to these non-disease events as phenotypes (e.g*.*, ‘abnormal cell proliferation’ is a phenotype while ‘lung cancer’ is a disease; ‘increased adipogenesis’ is a phenotype while ‘obesity’ is a disease). Curating phenotype data can provide information about chemical-induced events at the molecular and cellular level before a disease develops. To our knowledge, no other public database manually curates the scientific literature for the acquisition of chemical-induced (non-disease term) phenotypes. To that end, CTD biocurators reviewed 10 366 articles triaged for both a drug-of-interest and a phenotype from a list of 143 available terms preselected by Pfizer. To capture this data, CTD’s Curation Tool was modified to accommodate new phenotype action codes, 143 phenotype terms and 2774 anatomy terms (Figure 4A). From the drug–phenotype corpus, 36 742 phenotype interactions were curated, and an additional 1 341 interactions came from 401 articles of the drug–disease corpus that were incidentally curated for phenotype information during the transition period between projects (Table 2). In total, 9 489 curated articles yielded 38 083 phenotype interactions, of which 31 903 (84%) were for chemical–phenotype, 6% were for gene–phenotype and 10% were for complex chemical–gene–phenotype interactions (Figure 4B). Apoptosis was the most frequently curated phenotype, followed by blood pressure, cell proliferation, oxidative stress and cell cycle (Figure 2D).

Going forward, CTD plans to further develop and expand this phenotype module with a more comprehensive controlled vocabulary for non-disease terms frequently perturbed by chemicals. A candidate ontology is the GO-BP that contains over 25 700 terms and covers a greater range and granularity of biological events (25). CTD could easily transition this current pilot module to using GO. Seventy-five of the 143 MeSH phenotype terms used here already have direct equivalents in GO, and cross-mapping those terms retains 32 215 of the 38 083 (85%) curated interactions. This pilot data may help seed an expanded, fully integrated chemical–phenotype module in CTD.

### Phenotype–disease inferences

To demonstrate the utility of a curated phenotype dataset, we integrated the chemical–phenotype file with CTD’s chemical–disease dataset to generate inferences between phenotypes and diseases: if phenotype A is directly curated to chemical B, and chemical B is directly curated to disease C, then phenotype A is inferred to disease C (via shared chemical B). Network scores, which CTD has used to rank chemical–disease inferences (15), were similarly generated for each phenotype–disease relationship. In total, 102 828 inferences were established, linking 120 phenotypes to 2817 diseases, based on shared chemicals (Supplementary File 2). Top inferences based on the highest number of shared chemicals (and network scores) included phenotype–disease inferences between blood pressure–hypertension, heart rate–bradycardia, oxidative stress–drug-induced liver injury and apoptosis–acute kidney injury.

**Figure 5.**
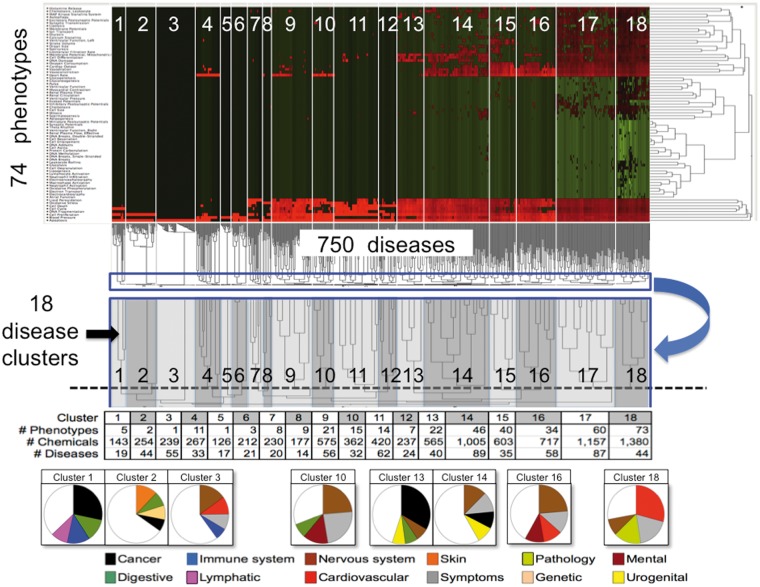
CTD phenotypes inferred to diseases through shared chemicals. A matrix of 74 phenotypes (rows) by 750 diseases (columns) was constructed where each cell represented the number of shared chemicals. The matrix was analysed by two-dimensional hierarchical clustering and visualized as a heatmap where the normalized number of shared chemicals are colored (green = low; black = medium; red = high). The similarities among the number of shared chemicals for diseases across all phenotypes are shown in the dendrogram beneath the heatmap, where the lengths of the lines are inversely proportional to the similarity (i.e., short = highly similar, long = dissimilar). An enlargement (blue boxes, blue arrow) shows how the disease dendrogram was trimmed to select 18 disease clusters (dotted line, with clusters numbered), and these boundaries are also represented on the heatmap (numbered white boxes). Below, the number of unique phenotypes, chemicals and diseases are charted for each cluster. In pie charts at the very bottom, predominant disease classes for some of the clusters are shown (only the top four disease classes are graphed). For example, of the 19 diseases in cluster 1, 28% of them represent cancers, 13% digestive system diseases, 13% immune system diseases and 9% lymphatic diseases. To the right of the heatmap, the similarities among the number of shared chemicals for phenotypes across all diseases are also shown in another dendrogram, where the lengths of the lines are inversely proportional to the similarity.

CTD then used this phenotype–disease inference file to construct a two-dimensional matrix, where each intersecting cell represented the number of shared chemicals between the phenotype and disease. For this analysis, inferences between a phenotype and disease were required to share a minimum of 10 chemicals. This stringency reduced the matrix to 74 phenotypes and 750 diseases. Two-dimensional hierarchical clustering ordered the phenotypes and diseases based on the similarity of the pattern profiles of shared chemicals (Figure 5).

Eighteen disease clusters were identified from the dendrogram. Many of the clusters show distinct disease classification profiles (pie charts, Figure 5). Cluster 1 has 5 phenotypes (cell cycle, proliferation, apoptosis, cell death and blood pressure) connected to 19 diseases; the largest disease class for this cluster is cancer, specifically of the immune, lymphatic and digestive systems (Figure 5). Interestingly, cluster 2 (which contains only apoptosis and cell proliferation) is connected to 44 diseases skewed toward a very different profile dominated by skin diseases (e.g., contact dermatitis, rosacea, localized scleroderma, erythema nodosum, etc.). Similarly, cluster 3 (which only contains apoptosis) includes 55 diseases, mostly of the nervous system and cardiovascular system. Preliminary analysis indicates the potential for making meaningful connections between chemicals, early pre-disease phenotypes and diseases.

### Text-mining precision

To measure the quality of the automatically extracted terms and events, we compared Pfizer’s text-mined terms supplied with each article (input) with the terms selected by CTD biocurators for curation (output). As input to biocurators, Pfizer text-mining queries (using their subsets of chemicals and diseases of interest) retrieved 1261 unique drug, 958 unique disease and 91 unique phenotype terms. Ultimately for output, biocurators curated to 5 562 unique chemical, 2 697 unique disease and 121 unique phenotype terms (adhering to CTD policy to curate all mentioned actors and not just the terms for which the article was triaged).

**Figure 6.**
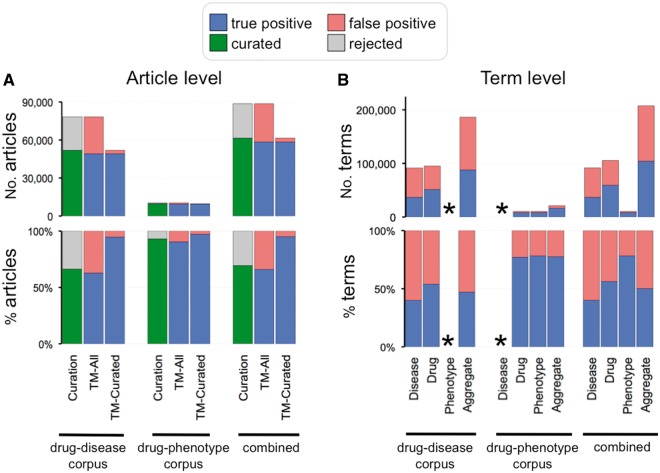
Curation and text-mining metrics. (**A**) Curation and text-mining metrics at the article level. The top graph shows the number of articles and the bottom graph shows the percentage for each corpus (drug–disease, drug–phenotype and combined). Curation metrics are measured by the number of curated articles (green bars) vs. number of rejected articles (gray bars). Text-mining metrics are measured by true positives (blue bars) vs. false positives (red bars) and measured against all the articles in the corpus (TM-All) as well as against solely the curated articles in the corpus (TM-Curated). (**B**) Text-mining metrics at the term level. The top graph shows the number of text-mined terms and the bottom graph shows the percentage for each term category (disease, drug, phenotype and aggregate of all the text-mined terms) from each corpus. Phenotype terms were not text mined for the drug–disease corpus and disease terms were not text mined for the drug–phenotype corpus (indicated by asterisks).

For the drug–disease corpus, 51 884 articles (66%) contained curatable information and 26 379 (34%) were rejected; for the drug–phenotype corpus, 9 646 articles (93%) were curated and a mere 720 (7%) were rejected; and combining the 2 sets, 69% of all the Pfizer articles were curated and 31% were rejected (Figure 6A). This overall rejection frequency was better than CTD's historic, pre-text mining rejection frequency of 40% (26).

To gauge text-mining effectiveness, we calculated the raw number and percentage of true positives (i.e., where an article’s input text-mined term, or child of that term, matched an output curated term) vs. false positives (i.e., where none of an article’s input terms, or children of those terms, matched any of the output curation). Metrics were calculated at both the article level and term level for the two separate collections (drug–disease and drug–phenotype) as well as the combined set.

#### Article level

For the 78 263 articles in the drug–disease corpus, 49 090 (63%) had true positives for any one matched chemical and/or disease term; however, this frequency increases to 95% when measured exclusively against the pool of 51 884 successfully curated articles for this corpus (Figure 6A). Likewise, for the 10 366 articles in the drug–phenotype corpus, 9 369 (90%) had true positives, and this frequency also increases to 97% when measured solely against the pool of 9 646 curated articles (Figure 6A). Lastly, when the 2 are combined, 58 459 articles (66%) had true positives, which increased to an aggregate percentage of 95% when only considering the pool of 61 530 curatable articles (Figure 6A).

#### Term level

For the drug–disease corpus, Pfizer identified 186 419 occurrences of text-mined input terms (94 996 occurrences for drugs and 91 423 for diseases). Of the 94 996 drug terms, 51 181 (54%) were true positives, and of the 91 423 disease terms, 36 624 (40%) were true positives (Figure 6B). For the drug–phenotype corpus, there were 20 904 occurrences of input terms (10 478 for drugs and 10 426 for phenotypes). Of the 10 478 drug terms, 8 077 (77%) were true positives; of the 10 426 phenotype terms, 8 157 (78%) were true positives (Figure 6B). Combining the sets, 40, 56 and 78% of all Pfizer text-mined disease, drug and phenotype terms were curated (respectively), producing an overall aggregate hit frequency of 50% for the entire project (Figure 6B).

The higher correlation between phenotype-based text mining (input) and ultimate curation (output) may be due to Pfizer and CTD using the same MeSH terms that the National Library of Medicine uses to index PubMed abstracts (27). The higher true positive frequency for the drug–phenotype corpus compared to the drug–disease corpus (77% vs. 54%, respectively) might be due to several factors. First, the semantic pattern used in the text mining for the former corpus did not allow more than two words between the bracketed concepts in ‘[DRUG] *regulatory verb phrase* [MeSH TERM]’. This restricted proximity between terms may have biased the corpus to only articles with the most direct drug–phenotype interactions, and hence resulted in both higher curation frequency (93%) as well as higher true positives for drug terms (77%) and phenotypes (78%). Second, CTD does not curate ‘negative’ data for chemical–disease interactions. Thus, if an article reported how a drug did not have an effect upon a disease, that information was not curated; however, ‘negative’ events were permissive for phenotype interactions, allowing biocurators to code interactions describing how drugs might inhibit or not affect a phenotype. Third, there was a 7.5-fold difference in sample size between the two sets of articles (10 366 vs. 78 263 articles, respectively).

Overall, these text-mining results were impressive when viewed in the context of the CTD curation process, because measuring the effectiveness of text mining at CTD can be understated. For example, there are many instances where cited text-mining terms are not actually involved in the types of interactions/relationships captured by CTD biocurators. Consequently, the complete universe of valid, cited text-mining terms specifically resident within each article is not necessarily recorded by CTD biocurators (28). One key metric that would seem to most accurately reflect the success of the Pfizer text mining is that 95% of the curatable articles contained one or more of the text-mined terms in an interaction. The increase in the text-mining success rate between all articles vs. curated articles (from 66% to 95%) suggest that the rejected articles more often than not contained the text-mined terms, just not in a context in which they were suitable for CTD curation.

## Summary

Text mining and manual curation of the scientific literature is a way to discover and unlock vast amounts of data originally stored as free-text by authors. Curating this data into structured formats via the use of controlled vocabularies and ontologies helps convert the information into computable knowledge, which can be more easily and accurately managed, queried, explored and analysed. Here, we described how a successful collaboration between Pfizer safety scientists and the biocuration staff at CTD resulted in the text mining and manual review of over 88 000 scientific articles to develop a dataset of drug-induced adverse events skewed toward cardiovascular, neurological, renal and hepatic toxicity.

**Figure 7.**
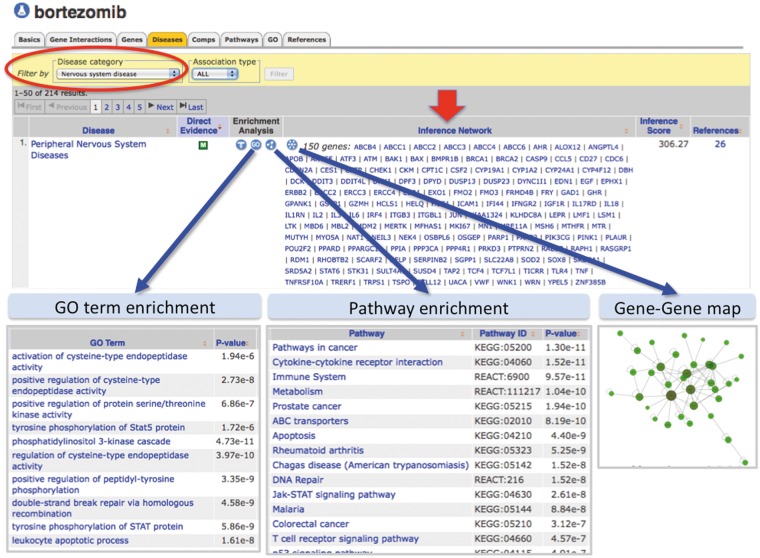
Enhanced content helps develop testable hypotheses for known drug–disease events. CTD’s page for the drug bortezomib is selected for ‘Diseases’ data (orange tab), and the results have been filtered for the category ‘Nervous system disease’ (red circle) to focus on NeuroTox events. Bortezomib is inferred to peripheral neuropathy by 150 genes (red arrow, ‘Inference Network’). Embedded web tools automatically generate lists of enriched GO terms, pathway annotations and gene–gene interaction maps (blue arrows).

This enhanced curated content can now be used to fill in the molecular gaps and find putative genes and pathways for developing testable hypotheses for drug–disease processes since CTD provides inference networks of genes that connect chemicals to diseases (11). For example, the drug bortezomib (a proteasome inhibitor used to treat multiple myeloma) is known to cause peripheral neuropathy in some patients, but the mechanistic process is still not clear (29). CTD discovers 150 genes that connect bortezomib to peripheral neuropathy, and the embedded web tools automatically calculate the enriched GO terms, pathway annotations and interaction maps for those connecting genes (Figure 7). This sophisticated knowledge management system can help researchers generate novel hypotheses about expanded molecular pathways of the drug–disease event and facilitate new screening assays for future pharmaceutical compound survival.

This curation is freely available to the public through CTD. As well, the data will be inevitably disseminated further into the scientific community via more than 50 other external databases that routinely incorporate CTD’s manual curation into their aggregated resources (http://ctdbase.org/about/publications/#use). In fact, curation from this project has already been incorporated into MetaADEDB, a new database of adverse drug events (30,31). As well, the dataset has been leveraged recently as a reference set to validate new algorithms for drug repositioning (32), as a standard for comparing successful drug–disease and drug–gene knowledge entity metrics (33), and as a resource for identifying chemical etiologies of diabetes (34). Additional improvements in text-mining strategies and manual biocuration will continue to enhance CTD as a premier resource for predictive toxicology. Other public–private relationships between database experts and commercial entities may also result in similar custom curation projects that can be shared with the scientific community.

## Citing and linking to CTD

To cite CTD, please see: http://ctdbase.org/about/publications/#citing. Currently, 53 external databases link to or present CTD data on their own websites. If you are interested in establishing links to CTD data, please notify us (http://ctdbase.org/help/contact.go) and follow these instructions: http://ctdbase.org/help/linking.jsp.

## Supplementary Data

Supplementary data are available at *Database* Online.

Supplementary Data
