# Supplementary material for: A CTD–Pfizer collaboration: manual curation of 88 000 scientific articles text mined for drug–disease and drug–phenotype interactions
Source: Database (Oxford). 2013 Nov 28;2013:bat080. doi: 10.1093/database/bat080 (PMC3842776; doi:10.1093/database/bat080)
Supplement: Supplementary Data [file supp_2013_bat080_index.html]

A CTD–Pfizer collaboration: manual curation of 88 000 scientific articles text mined for drug–disease and drug–phenotype interactions — Supplementary Data 

# A CTD–Pfizer collaboration: manual curation of 88 000 scientific articles text mined for drug–disease and drug–phenotype interactions

## Supplementary Data

files

**Files in this Data Supplement:**

- Supplementary Data - xls file
- Supplementary Data - xlsx file
